# Characterization of hairless (*Hr*) and *FGF5* genes provides insights into the molecular basis of hair loss in cetaceans

**DOI:** 10.1186/1471-2148-13-34

**Published:** 2013-02-09

**Authors:** Zhuo Chen, Zhengfei Wang, Shixia Xu, Kaiya Zhou, Guang Yang

**Affiliations:** 1 Jiangsu Key Laboratory for Biodiversity and Biotechnology, College of Life Sciences, Nanjing Normal University, Nanjing, 210023, China; 2 Present address: College of Life Sciences, Henan Normal University, Xinxiang, 453007, China

## Abstract

**Background:**

Hair is one of the main distinguishing characteristics of mammals and it has many important biological functions. Cetaceans originated from terrestrial mammals and they have evolved a series of adaptations to aquatic environments, which are of evolutionary significance. However, the molecular mechanisms underlying their aquatic adaptations have not been well explored. This study provided insights into the evolution of hair loss during the transition from land to water by investigating and comparing two essential regulators of hair follicle development and hair follicle cycling, i.e., the Hairless (*Hr*) and *FGF5* genes, in representative cetaceans and their terrestrial relatives.

**Results:**

The full open reading frame sequences of the *Hr* and *FGF5* genes were characterized in seven cetaceans. The sequence characteristics and evolutionary analyses suggested the functional loss of the *Hr* gene in cetaceans, which supports the loss of hair during their full adaptation to aquatic habitats. By contrast, positive selection for the *FGF5* gene was found in cetaceans where a series of positively selected amino acid residues were identified.

**Conclusions:**

This is the first study to investigate the molecular basis of the hair loss in cetaceans. Our investigation of *Hr* and *FGF5*, two indispensable regulators of the hair cycle, provide some new insights into the molecular basis of hair loss in cetaceans. The results suggest that positive selection for the *FGF5* gene might have promoted the termination of hair growth and early entry into the catagen stage of hair follicle cycling. Consequently, the hair follicle cycle was disrupted and the hair was lost completely due to the loss of the *Hr* gene function in cetaceans. This suggests that cetaceans have evolved an effective and complex mechanism for hair loss.

## Background

Hair is one of the distinguishing characteristics of mammals and it has many important biological functions [1]. Hair is produced by hair follicles (HFs), which are complex mini-organs in the skin that are formed during embryonic development (morphogenesis). New hair is generated continuously throughout life because the postnatal HFs experience cyclic phases of active growth (anagen stage), regression (catagen stage), and inactivity (telogen stage) [2]. Many genes and signaling pathways are involved in HF development [2,3], including the hairless (*Hr*) gene and fibroblast growth factor 5.

The *Hr* gene is significantly expressed in skin and it encodes a putative zinc finger transcription factor of approximately 130 kDa [4]. *Hr* is a candidate gene that regulates basic HF functions [5]. More detailed biochemical analyses of the function of the encoded protein have shown that *Hr* is a transcriptional corepressor that interacts with nuclear receptors, including thyroid hormone receptor (TR), retinoic acid orphan receptor α (RORα) and vitamin D receptor (VDR), to regulate specific target genes involved with hair morphogenesis and HF cycling [6,7]. It appears that *Hr* functions during the cellular transition to the first adult hair cycle because hair growth ceases completely in its absence, which results in a form of inherited total alopecia [8].

The fibroblast growth factor 5 (designated as *Fgf5* in rats and mice, and *FGF5* in other mammals), comprises three exons and it is an essential regulator of the HF development and cycling [9]. Recent studies have also suggested that the *FGF5* gene is associated with hair length and it controls the cessation of the anagen stage [2,10-13].

Cetaceans (whales, dolphins and porpoises) are ecologically diverse and they inhabit waters that range from coastal to oceanic and from tropical to polar [14]. Numerous paleontological, morphological, embryological, and molecular studies have suggested that cetaceans evolved from terrestrial mammals [15-19]. The transition from land to water and their subsequent adaptation to completely aquatic habitats make cetaceans remarkable and evolutionarily significant, although few studies have investigated the molecular basis of this process [20-26].

Cetaceans generally lack a coat of hair, although some cetacean species retain a few hairs on their face while the fetus has whiskers in others. This is probably an adaptation that reduces friction and improves locomotion. However, the precise molecular mechanisms underlying hair loss are unclear. Given the important roles of the *Hr* and *FGF5* genes during HF morphogenesis and HF cycling, the current study determined the full open reading frame (ORF) sequences of these two genes in seven representative cetacean species and compared them with orthologous sequences from terrestrial mammals. The goal was to determine whether evolutionary changes in these two genes were associated with the transition from land to water, and the hair loss of cetaceans during this adaptive process. To the best of our knowledge, this is the first study to investigate the molecular basis of hair loss in cetaceans.

## Results

### Hairless (*Hr*) and *FGF5* genes in cetaceans

**Figure 1:**
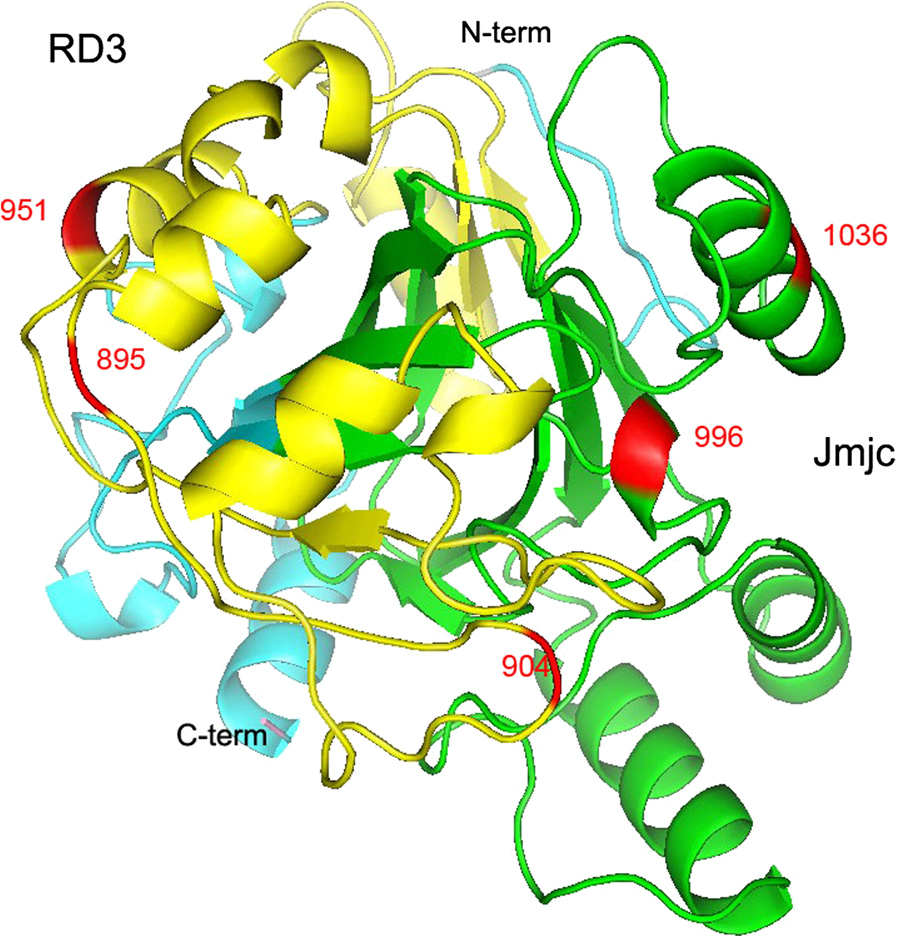
**Distribution of mutations in the three-dimensional structure of cetacean *Hr*.** The RD3 and JmjC domains are colored yellow and green, respectively. Locations marked in red coincide with mutations found within cetaceans and toothed whales within the RD3 and JmjC regions.

Complete ORF sequences were determined for the *Hr* and *FGF5* genes in seven cetaceans. As shown in Additional file 1, the cetacean *Hr* gene contained 18 exons and detailed information on each exon is provided in Table 1. They all shared the typical features of mammalian *Hr* genes and alignments of the deduced amino acid sequences of the cetacean *Hr* genes are shown in Additional file 2. No frame-shift mutations or premature stop codons were detected in cetaceans, although a series of apparent deletions and specific amino acid changes were found in important functional domains of the cetacean *Hr* genes (Figure 1, Additional files 2 and 3). In contrast to the toothed whale, the two baleen whales (*Balaenoptera omurai* and *B. acutorostrata*) lacked insertions/deletions (indels), so they had intact ORFs (Additional file 2).

**Table 1:** **Exon organization of the seven Cetacean *hairless *(*Hr*) genes obtained in this study**

**Exon**	**1**	**2**	**3**	**4**	**5**	**6**	**7**	**8**	**9**	**10**	**11**	**12**	**13**	**14**	**15**	**16**	**17**	**18**
*Tursiops truncatus*	612	784	151	188	165	90	116	82	164	219	166	70	131	120	116	165	129	63
*Delphinus capensis*	612	784	151	188	165	90	116	82	164	219	166	70	131	120	116	165	129	63
*Neophocaena phocaenoides*	612	784	151	188	165	90	116	82	164	213	166	70	131	120	116	165	129	63
*Delphinapterus leucas*	612	784	151	188	165	90	116	82	164	219	166	70	131	120	116	165	129	63
*Lipotes vexillifer*	612	784	151	188	165	90	116	82	164	237	166	70	131	120	116	165	129	63
*Balaenoptera omurai*	612	790	151	188	165	90	116	82	164	237	166	70	131	120	116	165	129	63
*Balaenoptera acutorostrata*	612	790	151	188	165	90	116	82	164	237	166	70	131	120	116	165	129	63

The cetacean *FGF5* also contained an uninterrupted ORF, with no premature stop codons. Three exons were identified based on an alignment of the cetacean *FGF5* sequences with those from other mammals. Exons 2 and 3 were highly conserved in all of the mammals examined in this study (Additional files 1 and 4). As shown in Additional file 4, the *FGF5* gene encoded a protein containing approximately 270 amino acid residues, including a signal peptide with 20 amino acid residues.

### Phylogenetic analysis

The maximum likelihood and Bayesian analyses of all datasets yielded similar tree topologies (Additional files 5 and 6). This strongly supported the nesting of Cetacea within Artiodactyla and the monophyly of Cetartiodactyla. Overall, the relationships among the nine placental mammalian orders examined were in agreement with those reported in previous studies, except for Perssodactyla (with horse as a representative) and the sister relationship between Odontoceti (toothed whales) and Mysticeti (baleen whales) within Cetacea (e.g., [27,28]).

### Relaxation of selection for cetacean *Hr* genes

**Figure 2:**
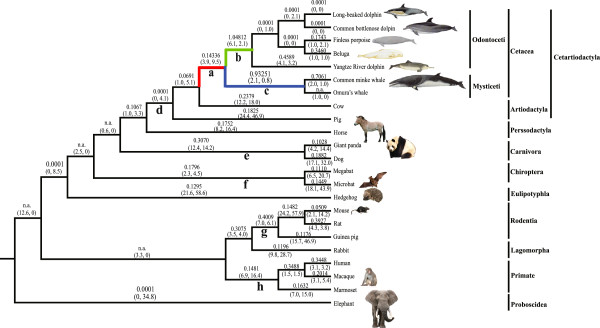
**The ω values of *FGF5 *genes in distinct evolutionary lineages of cetaceans and other mammals using a phylogenetic tree derived from Chen et al. **[29]**, Gatesy et al. **[27]**, and Zhou et al. **[19]**,**[28]**.** Branches a-h relate to those shown in Table 3. The ω values of individual branches shown are based on the free-ratio model. In some cases, zero synonymous substitutions produced an ω value of infinity (n.a.). The estimated numbers of nonsynonymous and synonymous changes are shown in parentheses. The ω values of branches a, b, and c (marked with different colors) were estimated using the two-ratio models.

A series of evolutionary models were performed with the likelihood framework to analyze the selective constraints on *Hr*, using the species tree shown in Figure 2 as the working topology. One-ratio model analyses of all mammals (dataset І: 17 sequences) showed that all of the branches in Figure 2 shared the same estimated ω of 0.2791 (Model A in Table 2), which indicated the existence of strong functional constraints on mammalian *Hr* genes. In the two-ratio model analyses (model B in Table 2), the ω value of the focal branch was 0.40432 and model B fitted the data significantly better than a one-ratio model, which assumed a single ω for all branches (*P* = 0.024, Table 2). A comparison of model B and model C (ω_2_ is fixed at 1, Table 2) also showed that ω_2_ was significantly less than 1, which suggested that the relaxation of the functional constraint on the *Hr* gene did not occur immediately after the common cetacean ancestor diverged from the terrestrial mammals.

**Table 2:** **Likelihood ratio tests using various models to determine the selective pressures on the cetacean *Hr *gene**

**Models**	**ω(*d***_***N***_**/*d***_***S***_**)**	**ln*L***^**a**^	**np**^**b**^	**Models compared**	**2Δ(ln*L*)**^**c**^	***P*-values**
**Dataset I: 17 sequences (16 terrestrial mammals plus the ancestral cetacean sequence)**						
A. All branches with the same ω	ω = 0.27908	−24914.624502	34			
B. Branch a (ancestral to cetacean) with ω_2_, other branches with ω_1_	ω_1_ = 0.27502, ω_2_ = 0.40432	−24912.059374	35	A vs B	5.130256	0.024
C. Branch a with ω_2_ = 1, other branches with ω_1_	ω_1_ = 0.27427, ω_2_ = 1	−24925.508371	34	B vs C	26.89799	2.14E-07
**Dataset II: 19 sequences (16 terrestrial mammals plus two baleen whales and the ancestral sequence of toothed whales)**						
D. All branches with the same ω	ω = 0.28065	−25530.203259	38			
E. Branch b (ancestral to toothed whale) with ω_2_, other branches with ω_1_	ω_1_ = 0.27911, ω_2_ = 0.48639	−25528.780477	39	D vs E	2.845564	0.092
F. Branch b with ω_2_ = 1, other branches with ω_1_	ω_1_ = 0.27890, ω_2_ = 1	−25530.928574	38	E vs F	4.296194	0.038
**Dataset III: 22 sequences (16 terrestrial mammals plus five toothed whales and the ancestral sequence of baleen whales)**						
G. All branches with the same ω	ω = 0.28417	−26239.723434	44			
H. Branch c (ancestral to baleen whale) with ω_2_, other branches with ω_1_	ω_1_ = 0.28182, ω_2_ = 0.62531	−26236.569206	45	H vs G	6.308456	0.012
I. Branch c with ω_2_ = 1, other branches with ω_1_	ω_1_ = 0.28170, ω_2_ = 1	−26237.557075	44	H vs I	1.975738	0.160

Note: a, ln L is the log-likelihood score; b, number of parameters; c, twice the difference in ln L between the two models compared.

Interestingly, both the common ancestors of toothed whales and baleen whales had a higher ω compared with other mammals (model E and H; see Table 2). In addition, the likelihood ratio test (LRT) results detected no significant difference in the estimates using the two-ratio model where ω was not fixed for the branch of the common ancestor of baleen whales and that where it was fixed to 1 (*P* = 0.160; model H vs. I in Table 2), which suggested that the *Hr* gene was close to selective neutrality in baleen whales. Furthermore, a series of sites were polymorphic in cetaceans whereas they were highly conserved (monomorphic) in other mammals (Additional file 3). Overall, these results suggest that the cetacean *Hr* gene has experienced a significant relaxation of selection during its evolution from terrestrial mammals and subsequent lineage diversification.

### Positive selection for the cetacean *FGF5* gene

In the branch-specific model analyses, the ω ratio calculated in the one-ratio model (M0) was 0.16615 (Table 3), which indicated a generally strong purifying selection for the mammalian *FGF5* gene, whereas only the lineage leading to the common ancestor of toothed whales (branch b in Figure 2) contained significant evidence of positive selection (2ΔL=5.93932, *P* = 0.0148). In addition, the lineage leading to the common ancestor of baleen whales (branch c in Figure 2) had a higher ω value (ω_1_ = 0.93251) than the background (ω_0_ = 0.16491), although the two-ratio models did not fit significantly better than model M0 (Table 3). Significant LRT statistics and positively selected sites were detected in the lineage leading to the common ancestor of toothed whales (branch b: 2ΔL=9.50702, *P* = 0.0086) in the branch-site model, whereas most lineages outside the cetaceans showed no evidence of positive selection (Table 3 and Figure 2).

**Table 3:** **Likelihood values and parameter estimates for the *FGF5 *gene**

**Models**	**ln L**^**a**^	**Estimate of parameters**	**2ΔL**^**b**^**(*P*-value)**	**Positively selected sites**
M0:one-ratio	−4450.77242	ω = 0.16615		
**Branch-specific models**				
**Branch a (ancestral Cetacea)**				
Two-ratio	−4450.74677	ω_0_ = 0.16656, ω_1_ = 0.14336	0.0513	
			(*P* = 0.8208)	
**Branch b (ancestral Odontoceti)**				
Two-ratio	−4447.80276	ω_0_ = 0.16253, ω_1_ = **1.04812**	**5.93932**	
			**(*P*= 0.0148)**	
**Branch c (ancestral Mysticeti)**				
Two-ratio	−4449.912614	ω_0_ = 0.16491, ω_1_= 0.93251	1.719612	
			(*P* = 0.189743)	
**Branch d (ancestral Cetartiodactyla)**				
Two-ratio	−4449.692636	ω_0_ = 0.16802, ω_1_ = 0.0001	2.159568	
			(*P* = 0.1416845)	
**Branch e (ancestral Carnivora)**				
Two-ratio	−4450.028289	ω_0_ = 0.16284, ω_1_ = 0.28977	1.488262	
			(*P* = 0.2224863)	
**Branch f (ancestral Chiroptera)**				
Two-ratio	−4450.762558	ω_0_ = 0.16637, ω_1_ = 0.14605	0.019724	
			(*P* = 0.8883106)	
**Branch g (ancestral Rodentia)**				
Two-ratio	−4450.367662	ω_0_ = 0.16377, ω_1_ = 0.33139	0.809516	
			(*P* = 0.3682634)	
**Branch h (ancestral Primates)**				
Two-ratio	−4450.765224	ω_0_ = 0.16585, ω_1_ = 0.17607	0.014396	
			(*P* = 0.9044964)	
**Branch-site models**				
Null	−4370.04122	p_0_ = 0.80258, p_1_ = 0.19742, ω_0_ = 0.05816, ω_1_ = 1		
**Branch a (ancestral Cetacea)**				
Alternative	−4370.04122	p_0_ = 0.80258, p_1_ = 0.19742, p_2a_ = 0, p_2b_ = 0, ω_0_ = 0.05816, ω_1_ = 1, ω_2_ = 1	0 (*P* = 1)	None
**Branch b (ancestral Odontoceti)**				
Alternative	−4365.28771	p_0_ = 0.46391, p_1_ = 0.12028, p_2a_ = 0.33019, p_2b_ = 0.08561, ω_0_ = 0.05123, ω_1_ = 1, ω_2_ = **2.03039**	**9.50702**	**K24E(0.945);P34Q(0.914);S68P(0.535);S74N(0.947);S84T(0.94)**
			**(*P*= 0.0086)**	
**Branch c (ancestral Mysticeti)**				
Alternative	−4368.231030	p_0_ = 0, p_1_ = 0, p_2a_ = 0.80784, p_2b_ = 0.19216, ω_0_ = 0.05877, ω_1_ = 1, ω_2_ = 1	3.62038	**M119T(0.777);V235A(0.937)**
			(*P* = 0.163623)	
**Branch d (ancestral Cetartiodactyla)**				
Alternative	−4370.041222	p_0_ = 0.80258, p_1_ = 0.19742, p_2a_ = 0, p_2b_ = 0,	0 (*P* = 1)	None
		ω_0_ = 0.05816, ω_1_ = 1, ω_2_ = 1		
**Branch e (ancestral Carnivora)**				
Alternative	−4370.041222	p_0_ = 0.80258, p_1_ = 0.19742, p_2a_ = 0, p_2b_ = 0,	0 (*P* = 1)	**186T(0.571)**
		ω_0_ = 0.05816, ω_1_ = 1, ω_2_ = 1		
**Branch f (ancestral Chiroptera)**				
Alternative	−4369.925398	p_0_ = 0.75669, p_1_ = 0.18750, p_2a_ = 0.04473, p_2b_ = 0.01108, ω_0_ = 0.05728, ω_1_ =1, ω_2_ =1	0.231644	**84T(0.736)**
			(*P* = 0.8906337)	
**Branch g (ancestral Rodentia)**				
Alternative	−4370.041222	p_0_ = 0.80258, p_1_ = 0.19742, p_2a_ =0, p_2b_ =0,	0 (*P* = 1)	**43G(0.621)**
		ω_0_ = 0.05816, ω_1_ = 1, ω_2_ = 1		
**Branch h (ancestral Primates)**				
Alternative	−4370.041222	p_0_ = 0.80258, p_1_ = 0.19742, p_2a_ = 0, p_2b_ = 0,	0 (*P* = 1)	None
		ω_0_ = 0.05817, ω_1_ = 1, ω_2_ = 1		

Note: a, ln L is the log-likelihood score; b, likelihood ratio test (LRT) to detect positive selection.

Five codons were shown to be under positive selection in the branch leading to the common ancestor of toothed whales (branch b in Figure 2) according to the branch-site model (Table 3), and four of these were shown to have undergone radical changes (Table 4). These positively selected amino acids did not correspond to residues known to interact with the FGF receptor (*FGFR*) and heparin (data not shown), but many of them were involved in or near a region rich in O-glycosylation and N-glycosylation sites, near the signal peptide region, or the *FGFR-*binding sites (Additional file 4).

**Table 4:** ***FGF5*candidate amino acid sites under positive selection identified in toothed whales**

**Amino acid position**	**PAML (branch-site model A)**	**Radical change**	**Conservative change**	**Change**
24	0.945	Lys-Glu	—	Charge, polarity, and volume
34	0.914	Pro-Gln	—	Polarity
68	0.535	Ser-Pro	—	Polarity
74	0.947	Ser-Asn	—	Polarity and volume
84	0.94	—	Ser-Thr	

## Discussion

### Relaxed selection for the cetacean *Hr* gene suggests its functional loss

**Figure 3:**
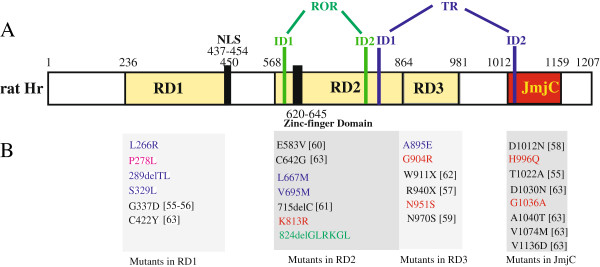
**Schematic representation of rat *Hr *structural and functional domains (reproduced from Thompson **[7]**; Hsieh et al. **[31]**) (A), and natural mutants of *Hr *that cause hair loss in mice and humans (B).** The positions of the three repression domains (RD1, RD2, RD3), and JmjC domain are indicated yellow and red, respectively. Nuclear localization signal (NLS), and zinc-finger domain are boxed in black [7,31]. Interacting domains 1 and 2 (ID1 and ID2) mediating the interaction between Hr and the retinoic acid receptor-related orphan receptor-alpha (RORα) [32] or with the thyroid hormone receptor (TR) [33] are boxed in green and blue, respectively. Specific cetacean, toothed whale, baleen whale, and Delphinoidea mutations are highlighted in red, blue, pink, and green, respectively [34-41].

The *Hr* gene is highly conserved and has traditionally been regarded as strongly functionally constrained during mammalian evolution because of its functional significance in HF cycling and the important roles of hair in mammals [1,5] (Additional file 3 and Table 2). However, our data suggest that the cetacean *Hr* gene may have experienced a relaxation of selective pressure to become a pseudogene (Table 2). Pseudogenes that experience relaxed selective pressure are expected to have a higher ω ratio compared with functional genes, which are usually under purifying selection [22]. Our analyses of the ω ratio based on different datasets showed that the ω ratios for the *Hr* sequences of toothed whales, baleen whales, and all cetaceans were clearly higher than those of putative functional sequences in other mammals (Table 2). More importantly, the presence of a series of polymorphic (as opposed to conservative or monomorphic) sites in cetaceans may be further evidence for the relaxed selection of cetacean *Hr* genes. In addition, most of the mutations in cetacean *Hr* genes were found in or near important functional domains or conserved regions, which was also supported by the homology modeling analysis of of cetacean *Hr* genes (Figure 1, and Additional file 3). For example, H996Q and G1036A mutations in the JmjC domain probably have led to the loss of histone demethylase activity and constitutive methylation, which would have promoted the transcriptional repression of *Hr-*interacting signaling pathways genes and ultimately hair loss [7,30]. This was corroborated by many natural mutants in the JmjC domain, which cause hair loss in mice and humans (Figure 3). Many mutations, including deletions, insertions, nonsense, missense and splice-sites, in the functional domains of the *Hr* genes from human patients or rodent models have also been reported to cause congenital atrichia (hair loss) (reviewed in [7]). Many, if not all, of the mutations (especially six base pair (bp) and 18-bp deletions in toothed whales) found in cetaceans are predicted to disrupt the local secondary structure and produce defects in HF regeneration, as found in humans and mouse models.

Furthermore, mutations leading to the absence of *Hr* function were found to differ to some extent in toothed and baleen whales, e.g., the 6-bp deletion in exon 2 was only present in toothed whales. Mutations in the cetacean *Hr* gene also exhibited a taxa-specific pattern. For example, the four delphinoids examined in this study all had an identical 18-bp deletion in exon 10. Another 6-bp deletion was identified in exon 10 of the finless porpoise. These different mutations in the *Hr* gene in different cetaceans may have resulted in a similar hair loss phenotype, which agrees with the fact that different mutations produce the same form of alopecia (hair loss) in humans and mice [7,42].

In summary, this study suggests that the cetacean *Hr* gene has undergone various evolutionary changes that probably correspond to its loss of function. These findings are consistent with morphological evidence that adult whales have no body hair covering, whereas they have hair during their early development [14]. During the transition from land to water, the ancestor of cetaceans was faced with different habitats and survival challenges, and the presence of hair may have been a hindrance to locomotion. Therefore, hair was probably unnecessary for cetaceans based on the cetacean *Hr* gene.

### Positive selection for the cetacean *FGF5* gene

In contrast to the relaxed selection for the *Hr* gene, the cetacean *FGF5* gene was under strong positive selection, according to the significantly higher ω value on the branch leading to the toothed whale compared with the background and the large number of specific codons detected by the branch-site models (Tables 3). The ω value (ω_1_ = 0.93251) of the lineage leading to the common ancestor of baleen whales was not significantly higher than 1, but this value was higher than the background value (ω_0_ = 0.16491) (Tables 3). This elevated ω estimate for the *FGF5* gene relative to other branches suggests the accelerated evolution of the *FGF5* gene in baleen whales. However, no evidence of positive selection was detected in the common ancestor of Cetacea (Tables 3), which suggests that positive selection occurred after the Odontoceti-Mysticeti split. Furthermore, a series of potentially important adaptive amino acid changes were detected in toothed whales (Table 3 and Additional file 4) and most of these codon changes had radical effects on their physicochemical properties (charge, polarity, and volume) (Table 4). In general, more radical amino acid substitutions have greater functional effects during evolution [43]. In addition, these residues were located in or near a region rich in O-glycosylation and N-glycosylation sites, near the signal peptide region or near the *FGFR-*binding sites. Therefore, these amino acid changes may have affected the secondary or tertiary conformation of the *FGF5* molecule and ultimately affected its function [44,45].

Accumulating evidence suggests that the *FGF5* gene controls the cessation of the anagen stage of the HF cycle and pathogenic mutations in this gene are known to produce long hair in phenotypic variants of many mammals [9,10,12,13,46]. It has been suggested that the epithelium and underlying mesenchyma interact in utero to form HFs (hair morphogenesis), before the HFs enter their three stages (anagen, catagen, and telogen) [3,47], which is consistent with embryological evidence that cetaceans develop body hair in the womb [17,48]. However, no extant whales retain any body hair after birth, with the exception of some snout hairs and hairs around the blowholes that act as sensory bristles in some baleen whales.

Humans and mice with mutations in the *Hr* gene develop apparently normal HFs but shed their hair completely soon after birth [4,42]. However, cetaceans lose their body hair in the womb rather than after birth. In addition, no positive selection was detected in *FGF5* genes in other branches, including the human and mouse genes. Overall, this study suggests that positive selection for *FGF5* genes in toothed whales may have played an important role in terminating the hair growth cycle and accelerated entry into the catagen stage of hair growth. Interestingly, the current study showed that the *Hr* gene may have lost its function in cetaceans. This gene loss initiates a premature and abnormal catagen stage, which leads to the destruction of the normal HF architecture and abrogates the HF’s ability to cycle. This hypothesis may be tested by elucidating the molecular mechanism of hair loss in cetaceans and the differences in hair loss between cetaceans and *Hr* mutants in humans and mice. However, it is not easy to obtain cetacean biopsies for expression analyses, and so further research (e.g., expression experiment or histochemistry) will be required to test our hypotheses of *Hr* pseudogenisation and *FGF5* positive selection in cetaceans. Further samples should also be obtained from cetaceans (especially baleen whales) and other fully aquatic marine mammals (e.g., sirenians: manatees and dugongs) to test this hypothesis and elucidate the molecular basis of hair loss.

## Conclusions

The data presented in this study suggest that the cetacean *Hr* gene has undergone evolutionary changes related to its loss of function. By contrast, positive selection on the *FGF5* gene was detected in cetaceans, including a series of positively selected amino acid residues. The evolutionary changes in these two genes may provide new insights into the molecular basis of significant hair loss in cetaceans during their transition from land to water. Many signaling pathways and factors are known to be involved in the regulation of HF morphogenesis and the HF cycle [2,3]. Therefore, additional genes related to hair development should be investigated to improve our understanding of the molecular mechanism underlying hair loss in fully aquatic marine mammals.

## Methods

### Taxonomic coverage

Seven cetacean species (five odontocetes and two mysticetes) were sequenced during this study (Table 5). In addition, the full-length ORFs of *FGF5* and *Hr* from 16 other mammals were searched and downloaded from the Ensemble Genome database (http://www.ensembl.org) and GenBank (http://www.ncbi.nlm.nih.gov) (Table 5). When multiple splice vari-ants of *FGF5* and *Hr* were available, only the full-length form from a species was used in subsequent analyses.

**Table 5:** List of taxonomic samples and sequences used in this study

**Order**	**Suborder**	**Species**			**Accession number**	
		**Family**	**Scientific name**	**Common name**	***Hr***	***FGF5***
**Cetacea**	**Odontoceti**	**Delphinidae**	*Tursiops truncatus*	Common bottlenose dolphin	KC140206	KC140213
			*Delphinus capensis*	Long-beaked common dolphin	KC140205	KC140212
		**Monodontidae**	*Delphinapterus leucas*	Beluga or white whale	KC140208	KC140215
		**Phocoenidae**	*Neophocaena phocaenoides*	Finless porpoise	KC140207	KC140214
		**Lipotidae**	*Lipotes vexillifer*	Yangtze river dolphin or baiji	KC140209	KC140216
	**Mysticeti**	**Balaenopteridae**	*Balaenoptera acutorostrata*	Common minke whale	KC140210	KC140217
			*Balaenoptera omurai*	Omura's whale	KC140211	KC140218
**Artiodactyla**		**Bovidae**	*Bos taurus*	Cow	Genome sequence	Genome sequence
		**Suidae**	*Sus scrofa*	Pig	Genome sequence	Genome sequence
**Perissodactyla**		**Equidae**	*Equus caballus*	Horse	Genome sequence	Genome sequence
**Carnivora**		**Canidae**	*Canis familiaris*	Dog	Genome sequence	Genome sequence
		**Ursidae**	*Ailuropoda melanoleuca*	Giant panda	XM_002914796.1	XM_002912480.1
**Chiroptera**		**Pteropodidae**	*Pteropus vampyrus*	Megabat	Genome sequence	Genome sequence
		**Vespertilionidae**	*Myotis lucifugus*	Microbat	Genome sequence	Genome sequence
**Eulipotyphla**		**Erinaceidae**	*Erinaceus europaeus*	Hedgehog	Genome sequence	Genome sequence
**Rodentia**		**Muridae**	*Mus musculus*	Mouse	Genome sequence	Genome sequence
			*Rattus norvegicus*	Rat	Genome sequence	Genome sequence
		**Caviidae**	*Cavia porcellus*	Guinea pig	Genome sequence	Genome sequence
**Lagomorpha**		**Leporidae**	*Oryctolagus cuniculus*	Rabbit	Genome sequence	Genome sequence
**Primates**		**Hominidae**	*Homo sapiens*	Human	Genome sequence	Genome sequence
		**Cercopithecidae**	*Macaca mulatta*	Macaque	Genome sequence	Genome sequence
		**Callitrichidae**	*Callithrix jacchus*	Marmoset	Genome sequence	Genome sequence
**Proboscidea**		**Elephantidae**	*Loxodonta africana*	Elephant	Genome sequence	Genome sequence

### Amplification and sequencing of cetacean *FGF5* and *Hr* genes

Primers were designed for the conserved regions based on an alignment of genomic data from the cow *Bos taurus* (http://asia.ensembl.org/Bos_taurus/Info/Index) and bottlenose dolphin (*Tursiops truncatus*) (http://asia.ensembl.org/Tursiops_truncatus/Info/Index). The primer information is available upon request. Total genomic DNA was extracted from the muscle tissues using a standard phenol/chloroform procedure followed by ethanol precipitation [49]. For blood, we used the DNAeasy Blood Extraction Kit (Qiagen) in a separate laboratory facility. All PCR amplification were conducted using a BioRAD PTC-200 with 2×EasyTaq PCR SuperMix (TransGen Biotech) and the following profile: 34 cycles at 94°C for 5 min, 94°C for 30 s, 53°C −59°C for 30 s, and 72°C for 30 s, followed by a 10 min extension at 72°C. The amplified PCR products were purified and sequenced in both directions using an ABI 3730 automated genetic analyzer. Novel sequences were deposited in GenBank under accession numbers KC140205-KC140218.

### Sequence alignment and statistical analyses

Nucleotide sequences from the coding regions of *Hr* and *FGF5*, and their deduced amino acid sequences, were aligned separately using the program CLUSTAL X [50] and manually adjusted with GeneDoc. The nucleotide sequence alignment was generated based on the protein sequence alignment. Ancestral sequences were reconstructed using Bayesian method [51], which was implemented in the BASEML program in PAML 4.5 [52]. A three-dimensional domain structure of the cetacean *Hr* was predicted using SWISS-MODEL (http://swissmodel.expasy.org) [53-55].

Phylogenetic trees were reconstructed using maximum likelihood algorithms in MetaPIGA 2.0 [56] and Bayesian inference (BI) in MrBayes 3.1.2 [57] for each gene independently and for a combined dataset of *FGF5* and *Hr*, using the African elephant (*Loxodonta africana*) as the outgroup. MRMODELTEST 2.3 [58] was used to select the optimal models for each partition based on the Akaike Information Criterion (AIC). Maximum Likelihood analyses were performed using MetaPIGA 2 [56] with 1000 replicate metaGA searches. The Bayesian analyses of the nucleotide matrix were performed using a codon model (general time irreversible (GTR) gamma invariant model) or mixed models (the GTR gamma invariant model for the first and second codon positions, and the GTR gamma model for the third codon position). Four Markov chains were run for 20 million generations in MrBayes 3.1.2, with sampling every 1000 generations. The stationarity of the likelihood scores of the sampled trees were checked using Tracer 1.4 [59]. The Bayesian posterior probabilities (PP) were obtained from the 50% majority rule consensus of the post burn-in trees sampled at stationarity, after removing the first 10% of trees as a “burn-in” stage. The aligned sequences and phylogenetic trees were deposited in TreeBase (http://purl.org/phylo/treebase/phylows/study/TB2:S13758).

### Analysis of selective pressure

Analyses of selective pressure were carried out using a codon-based maximum likelihood method implemented in the CODEML program in the PAML 4.5 package [52]. A consensus tree that included all of the species employed in the present study was inferred from Chen et al. [29], Gatesy et al. [27], and Zhou et al. [19,28], and used in the subsequent PAML analyses (Figure 2). In all PAML-based analyses, the alignment gaps were treated as ambiguous characters (setting: cleandata = 0). All models corrected the transition/transversion rate and codon usage biases (F3×4). Different starting ω values were also used to avoid local optima on the likelihood surface [60].

Three codon substitution models of maximum likelihood analysis were produced to detect selective pressure acting on the *Hr* and *FGF5* genes: a site model, branch model, and branch-site model [61,62]. The branch-specific models permitted variable ω ratios among branches but invariable ω ratios in the sites in the tree and they could be implemented to study changes in selective pressures in specific lineages [63]. In the branch-specific models, a ‘one-ratio’ model (M0) that assumed the same ω ratio for all branches [64] was compared with models where ω was allowed to differ in the background and a focal branch (two-ratio model). The branch-site models permitted the ω ratio to vary among sites and among lineages, which was useful for detecting positive selection that affected only a few sites in a few lineages [63,65].

Significant for differences between two nested models were detected by using LRTs to calculate twice the log-likelihood (2ΔL) difference following a chi-square distribution, where the number of degrees of freedom was equal to the difference in the numbers of free parameters between models.

## Competing interests

The authors declare that they have no competing interests.

## Authors' contributions

GY and ZC designed the study. ZC and ZFW carried out the experiments, performed the data analyses, and prepared the draft of the manuscript. SX and KZ helped to improve the manuscript. GY helped to perform the data analyses and improve the manuscript. All authors read and approved the final manuscript.

## Supplementary Material

Additional file 1 **Gene structures of cetacean *Hr *(A) and *FGF5 *(B).** The exons are shown in black boxes and intron sizes shown in the figure are not proportionally scaled on both (A) and (B) because of the large size of the introns. Click here for file

Additional file 2 **Multiple sequence alignments of the newly obtained seven cetacean *Hr * deduced amino acids.** The positions of the three repression domains (RD1, RD2, RD3), nuclear localization signal (NLS), zinc-finger domain and JmjC domain are indicated [7,31]. Interacting domains 1 and 2 (ID1 and ID2) mediating the interaction between *Hr* and the retinoic acid receptor-related orphan receptor-alpha (RORα) [32] or with the thyroid hormone receptor (TR) [33] are boxed. Cysteine residues involved in formation of the potential zinc finger are highlighted in blue. Deletions and mutations of the five toothed whales are highlighted in yellow and red, respectively. Click here for file

Additional file 3 **Multiple sequence alignments of the newly obtained seven cetacean *Hr *deduced amino acids with other mammals in GenBank and Ensemble Genome database.** Specific mutations and polymorphic sites of cetaceans compared with the other terrestrial mammals are highlighted in red and green, respectively. The functional domains are indicated as in Additional file 2. Click here for file

Additional file 4 **Alignment of FGF5 amino acid sequences determined for the seven cetaceans in this study and other terrestrial mammals in GenBank and Ensemble Genome database.** The signal peptide and the glycine box are highlighted in blue and green, respectively. Black triangles depict O-glycosylation sites and a green vertical arrow is used to depict the N-glycosylation site. The *FGF* receptor (FGFR) and the heparin binding sites are indicated with red circles and red triangles, respectively [44]. Amino acid residues under positive selection in toothed whales and baleen whales are highlighted in red and yellow, respectively. Click here for file

Additional file 5 **Phylogenetic trees reconstructed using BI and ML methods based on dataset of *Hr*.** Integers associated with branches are MetaPIGA support values from ML analyses of *Hr* gene whereas values of 1 or less are Bayesian posterior probabilities. The relevant references in *FGF5* analysis are not shown. Click here for file

Additional file 6 **Phylogenetic trees reconstructed using BI and ML methods based on the concatenated dataset of *Hr *and *FGF5*.** Integers associated with branches are MetaPIGA support values from ML analyses whereas values of 1 or less are Bayesian posterior probabilities. Click here for file
