# Supplementary figures and images for: Characterization of hairless (Hr) and FGF5 genes provides insights into the molecular basis of hair loss in cetaceans
Source: BMC Evol Biol. 2013 Feb 9;13:34. doi: 10.1186/1471-2148-13-34 (PMC3608953; doi:10.1186/1471-2148-13-34)

**A.**

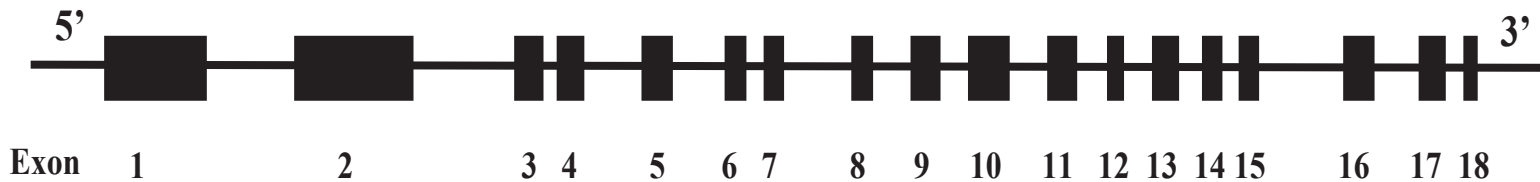

**B.**

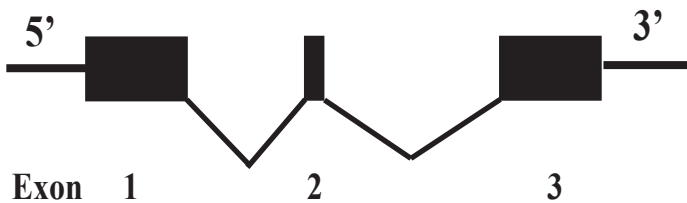

Supplement: Additional file 1 — Gene structures of cetacean Hr (A) and FGF5 (B). The exons are shown in black boxes and intron sizes shown in the figure are not proportionally scaled on both (A) and (B) because of the large size of the introns. [file 1471-2148-13-34-S1.pdf]

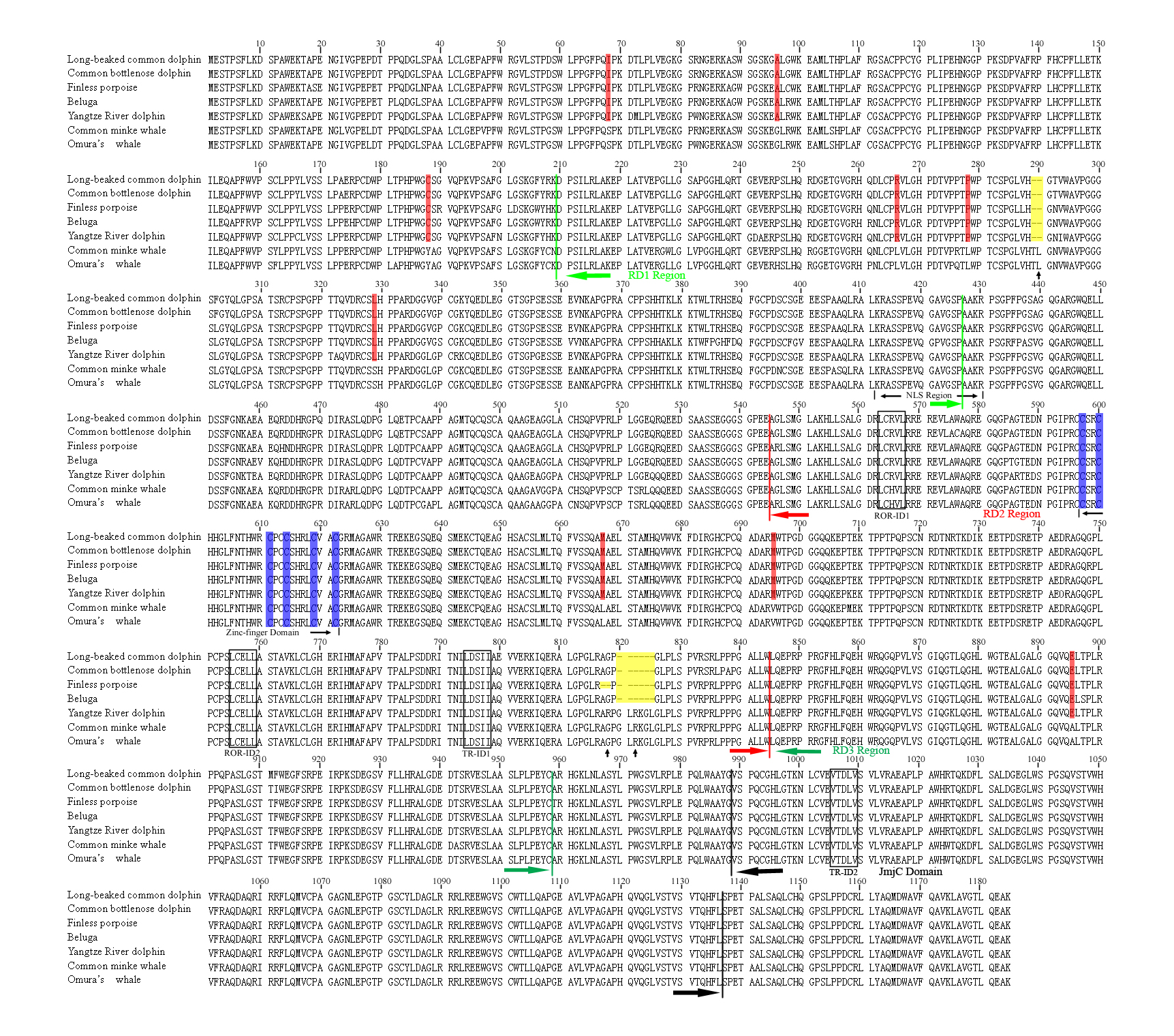

Supplement: Additional file 2 — Multiple sequence alignments of the newly obtained seven cetacean Hr deduced amino acids. The positions of the three repression domains (RD1, RD2, RD3), nuclear localization signal (NLS), zinc-finger domain and JmjC domain are indicated [7,31]. Interacting domains 1 and 2 (ID1 and ID2) mediating the interaction between Hr and the retinoic acid receptor-related orphan receptor-alpha (RORα) [32] or with the thyroid hormone receptor (TR) [33] are boxed. Cysteine residues involved in formation of the potential zinc finger are highlighted in blue. Deletions and mutations of the five toothed whales are highlighted in yellow and red, respectively. [file 1471-2148-13-34-S2.jpeg]

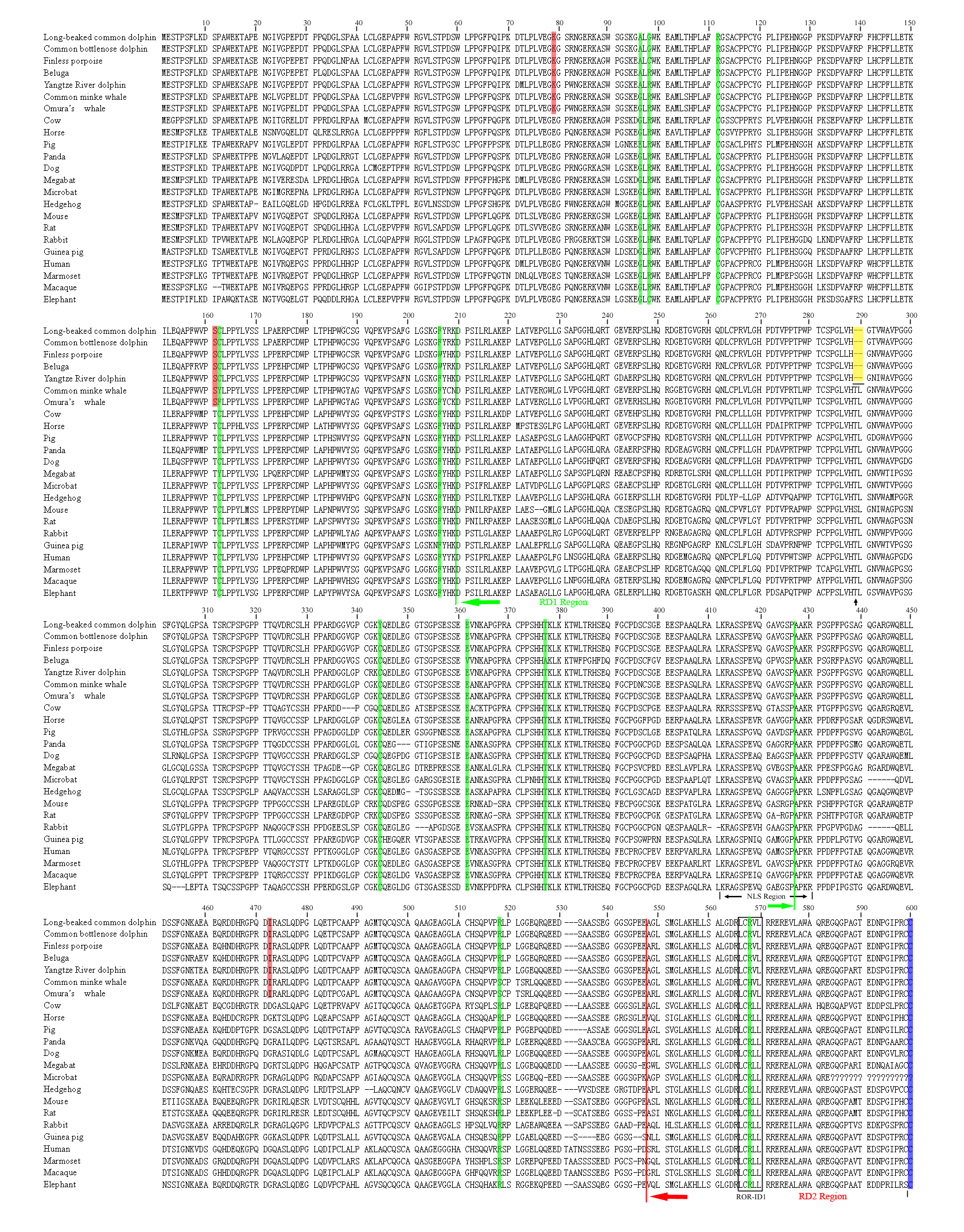


(continued)


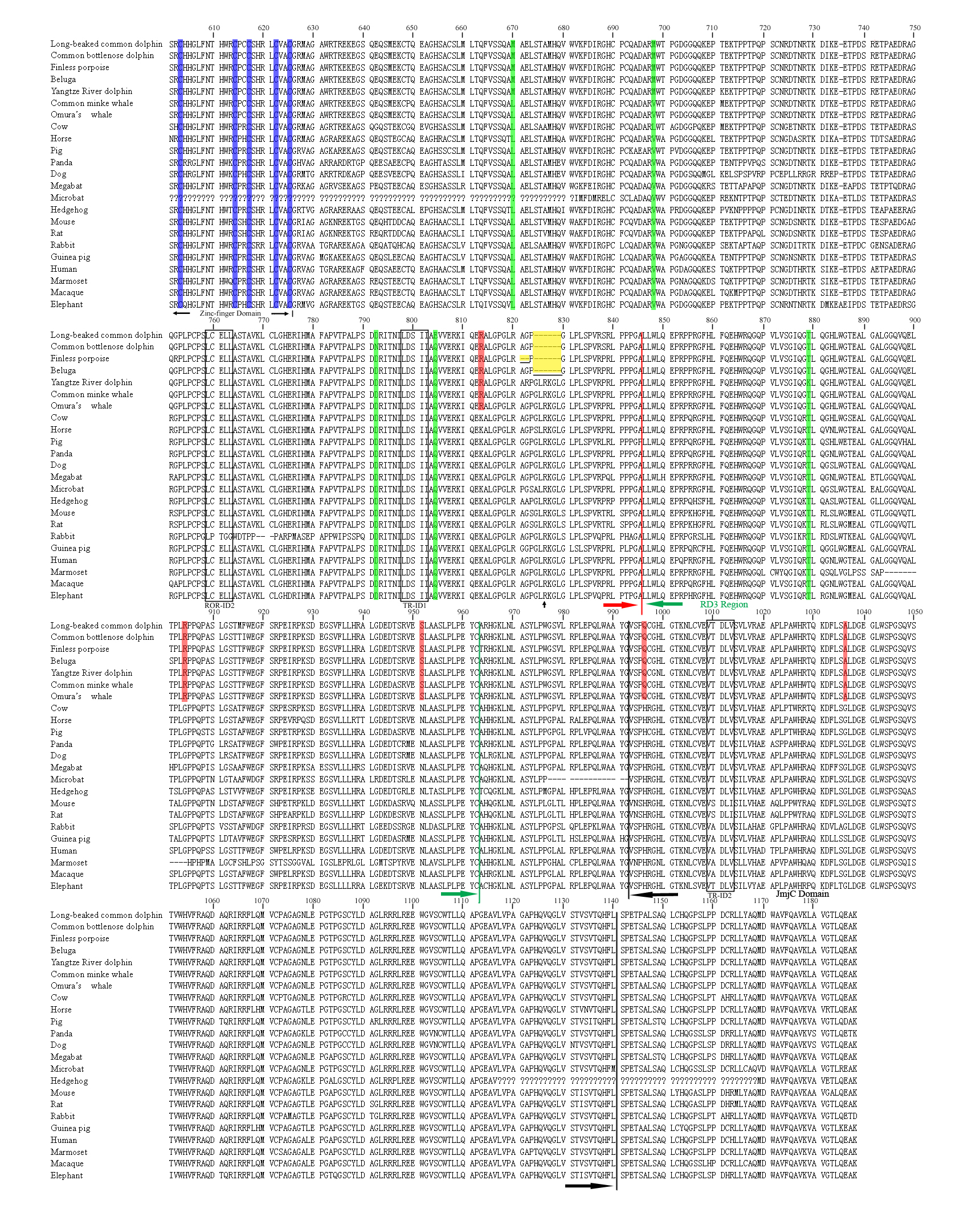

Supplement: Additional file 3 — Multiple sequence alignments of the newly obtained seven cetacean Hr deduced amino acids with other mammals in GenBank and Ensemble Genome database. Specific mutations and polymorphic sites of cetaceans compared with the other terrestrial mammals are highlighted in red and green, respectively. The functional domains are indicated as in Additional file 2. [file 1471-2148-13-34-S3.doc]

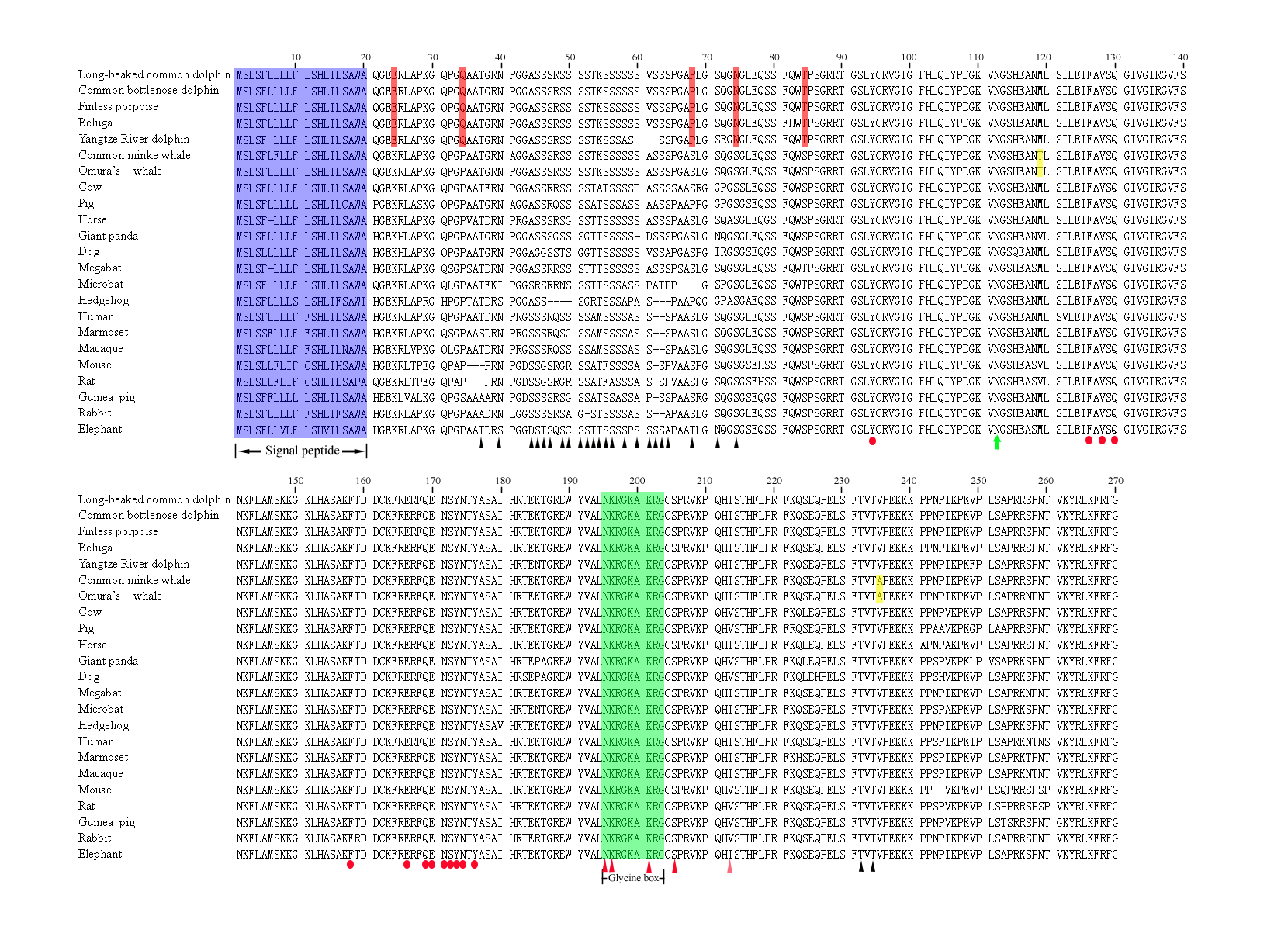

Supplement: Additional file 4 — Alignment of FGF5 amino acid sequences determined for the seven cetaceans in this study and other terrestrial mammals in GenBank and Ensemble Genome database. The signal peptide and the glycine box are highlighted in blue and green, respectively. Black triangles depict O-glycosylation sites and a green vertical arrow is used to depict the N-glycosylation site. The FGF receptor (FGFR) and the heparin binding sites are indicated with red circles and red triangles, respectively [44]. Amino acid residues under positive selection in toothed whales and baleen whales are highlighted in red and yellow, respectively. [file 1471-2148-13-34-S4.jpeg]

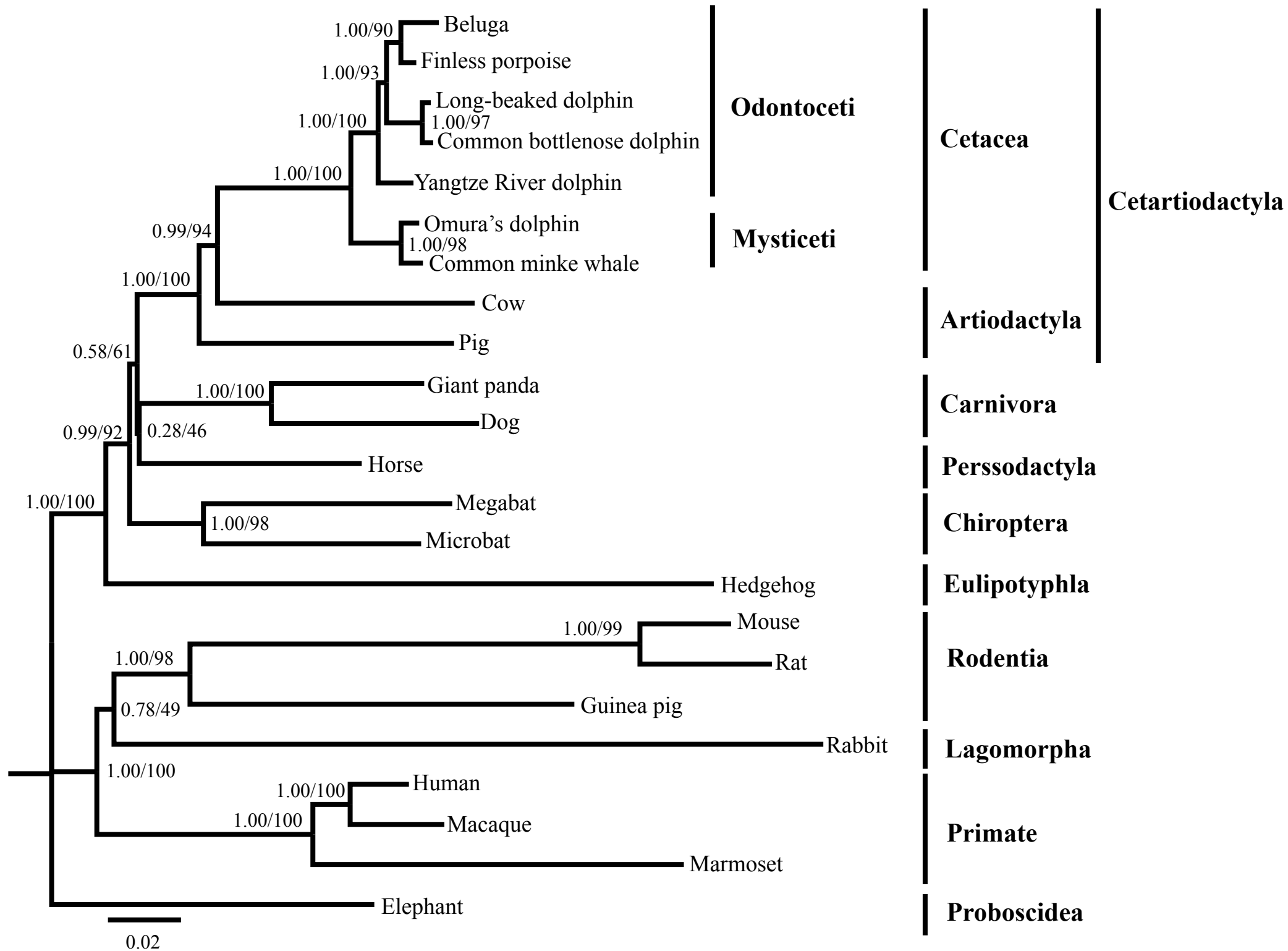

Supplement: Additional file 5 — Phylogenetic trees reconstructed using BI and ML methods based on dataset of Hr. Integers associated with branches are MetaPIGA support values from ML analyses of Hr gene whereas values of 1 or less are Bayesian posterior probabilities. The relevant references in FGF5 analysis are not shown. [file 1471-2148-13-34-S5.pdf]

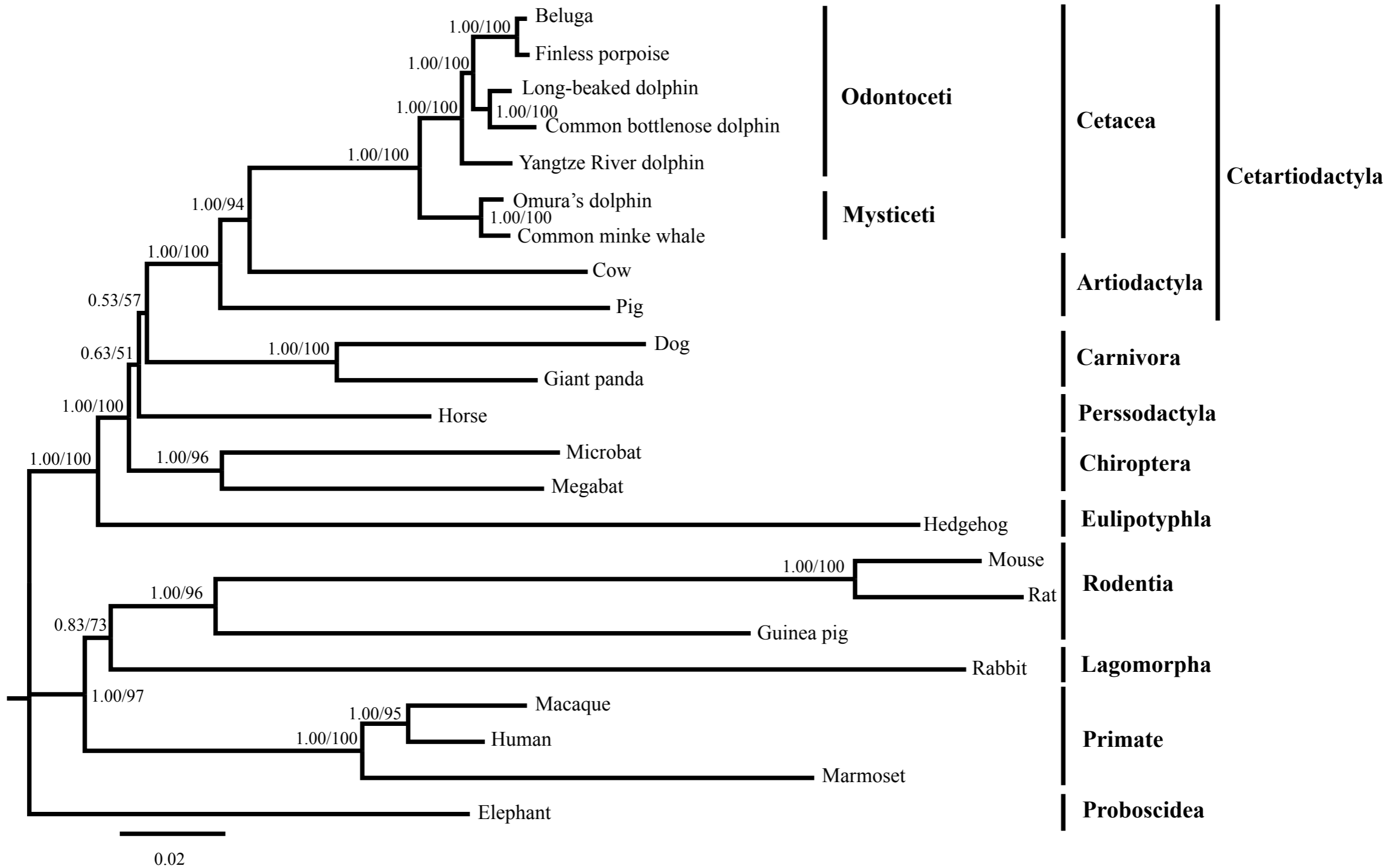

Supplement: Additional file 6 — Phylogenetic trees reconstructed using BI and ML methods based on the concatenated dataset of Hr and FGF5. Integers associated with branches are MetaPIGA support values from ML analyses whereas values of 1 or less are Bayesian posterior probabilities. [file 1471-2148-13-34-S6.pdf]
